# Combined Effects of Beetle Density and Sex Ratio on Reproductive Trade‐Offs and Dung Burial in Tunnelling Dung Beetles

**DOI:** 10.1002/ece3.74499

**Published:** 2026-10-16

**Authors:** Zinat Jahan Chowdhury, Zac Hemmings, Alfonsina Arriaga‐Jimenez, Karl Vernes, Timothy M. Schaerf, Nigel R. Andrew

**Affiliations:** ^1^ School of Environmental and Rural Science University of New England Armidale New South Wales Australia; ^2^ Department of Entomology Sylhet Agricultural University Sylhet Bangladesh; ^3^ Department of Primary Industries Department of Regional NSW Coffs Harbour New South Wales Australia; ^4^ Queen Victoria Museum and Art Gallery Launceston Tasmania Australia; ^5^ School of Science and Technology University of New England Armidale New South Wales Australia; ^6^ Entomology Lab, Faculty of Science and Engineering Southern Cross University Lismore New South Wales Australia

**Keywords:** brood, *Digitonthophagus gazella*, dung removal, ecosystem services, *Onthophagus binodis*, reproduction

## Abstract

Dung beetle density and sex ratio play a significant role in intraspecific competition, and reproductive success depends on the beetle's maturity. We examined the impact of increasing density in small groups on dung burial and brood production using mature first‐generation individuals of two dung beetle species ( *Onthophagus binodis* and *Digitonthophagus gazella*) by manipulating the number of males and females in a dung pat. Beetles were reared under controlled laboratory conditions and provided with cattle dung, which was collected from pastureland. Weekly dung burial was calculated by measuring the residual dung organic matter content. The number of broods produced per dung pat, broods' dry weight, and their size were recorded. In both species, treatments with intermediate numbers of male and female beetles produced more broods than solitary beetles. On the other hand, treatments with moderate to high numbers of male and female beetles buried more dung than individual females. At higher‐density treatments, brood size and brood dry weight declined, suggesting shifts in reproductive allocation under increasing intraspecific interactions. The two species showed very similar patterns, although *O. binodis* produced more broods while *D. gazella* buried more dung. We also observed a positive relationship between brood dry weight and brood size in both species. Overall, the results indicate that the combined effects of beetle density and sex ratio influenced reproductive investment and dung burial under controlled laboratory conditions. Further field studies are needed to determine whether similar patterns occur under higher density and natural ecological conditions.

## Graphical abstract

Moderate intraspecific density and sex ratio increased brood production in both *Onthophagus binodis* and *Digitonthophagus gazella*. Moderate to high numbers of male and female beetles buried the highest amount of dung compared with solitary beetles but produced smaller and lighter broods in both species. *Onthophagus binodis* prioritised brood number, whereas *D. gazella* maintained greater investment per brood across treatments, indicating species‐specific reproductive strategies.

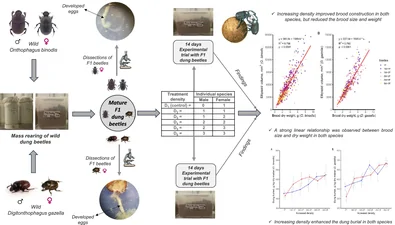

## 1 Introduction

Competition for limited resources is a fundamental ecological process that influences insect growth, development and reproduction (Boggs 2009). As population density increases, exploitative competition generally intensifies, reducing per‐capita resource availability and altering reproductive investment through trade‐offs between offspring number and quality (Agnew et al. 2002). Dung beetles (Coleoptera: Scarabaeidae) provide an excellent system for examining density‐dependent competition because they rely on dung resources, both in the laboratory and the field (Finn and Gittings 2003). Competition for limited resources can influence access to breeding resources, reproductive allocation and offspring performance, making dung beetles a useful model for understanding how resource limitation shapes reproductive strategies (Teixeira and Zucoloto 2012).

Dung beetles are important ecosystem engineers that primarily utilise vertebrate dung for feeding and reproduction while contributing to dung removal, bioturbation, soil aeration, nutrient cycling, secondary seed dispersal and fly control (Heddle et al. 2021; Nichols et al. 2008). Generally, dung beetles have three nesting strategies, each with ecological implications. Among them, paracoprid (tunnelling) beetles bury dung beneath the soil to construct brood, directly linking their reproductive behaviour with ecosystem functioning (Doube and Marshall 2014; Hanski and Cambefort 1991b). The current study focused on two widely established tunnelling dung beetles in Australian grazing systems, *Onthophagus binodis* ( *O. binodis* ) and *Digitonthophagus gazella* (*D. gazella*). Both species commonly coexist in cattle pastures, reproduce by burying dung in underground brood chambers, and differ in life‐history characteristics that may influence competitive interactions and resource allocation (Cook 1988; Edwards 2009; Pokhrel et al. 2021).

Intraspecific competition does not always involve direct interference among individuals but can also arise through density‐dependent constraints on resource allocation. In dung beetles, increasing density intensifies competition for limited dung resources, which can reduce individual fitness, alter reproductive investment, and influence dung burial (Hanski and Cambefort 1991a; Scholtz et al. 2009). Previous studies have shown that dung beetles at higher densities are associated with reduced brood production, lower dung burial and species‐specific variation in reproductive output (Holter 1979; Macqueen and Beirne 1975; Simmons and Ridsdill‐Smith 2011; Yasuda 1987). Collectively, these studies demonstrate that density influences reproductive and ecological performance, but most have focused on wild‐caught single‐species populations. Consequently, how the combined effects of density‐dependent intraspecific interaction and sex ratio shape reproductive investment and ecosystem functioning under controlled experimental conditions remains poorly understood.

Most density‐dependent studies on dung beetle competition have relied on field‐collected adults, with unknown ages and reproductive statuses (Giller and Doube 1989; Gotcha et al. 2022; Mamantov and Sheldon 2023). Although these studies have provided important ecological insights, age, reproductive maturity and body condition of beetles are recognised sources of variation in reproductive performance (Cook 1988; Dhillon et al. 2019; Kotiaho 2002). Consequently, differences in individual physiology may contribute to variation in ecological functions and reproductive output independent of experimental treatments (Correa et al. 2025). To minimise such potential confounding effects, we used laboratory‐reared first‐generation (F1) beetles of known age and confirmed reproductive maturity. In addition to beetle maturity and density, the sex ratio within breeding groups can influence both ecosystem engineering functions and reproductive performance in beetles, as male–female interactions affect dung burial behaviour and brood production (Zamprogna et al. 2025). Therefore, variation in sex composition was considered an important component of the study design for this work when evaluating density‐dependent intraspecific competition and reproductive outcomes.

We hypothesised that increasing intraspecific density in combination with changes in sex ratios would influence reproductive investment and ecosystem functioning of dung beetles. We investigated how increasing density and sex ratio affected (i) dung burial as a measure of ecosystem functioning, (ii) brood production as an indicator of reproductive output and (iii) brood volume and brood dry weight as measures of maternal investment per offspring. In addition, we examined the relationship between brood volume and brood dry weight across treatments to determine whether changes in brood size were associated with variation in resource allocation to developing offspring.

## 2 Materials and Methods

Ethical approval was not required for this study, as it involved only invertebrate species and did not include any procedures subject to Australian animal ethics regulations.

The experimental study was conducted using two introduced dung beetle species ( *O. binodis* and *D. gazella*) in a controlled‐temperature laboratory located in northern New South Wales, Australia. Experimental containers were maintained in a purpose‐built, centrally controlled laboratory room at approximately 25°C and 40% relative humidity. Temperature and relative humidity were regulated by Munters MLT800 PLUS air‐handling units, supplied by a Carrier 30RBS chiller and Chappée XRE409 gas‐fired boilers with Saacke PAG10B2 burners, and controlled through the Honeywell EBI Building Management System. Environmental conditions were monitored through the integrated room monitoring system. The laboratory lighting was automatically programmed to provide a 12 h light and 12 h dark photoperiod throughout the experiment. Both *O. binodis* and *D. gazella* are paracoprid (tunnelling) dung beetles in which males and females cooperate in dung burial and brood construction. The same environmental conditions were used for both species to standardise rearing conditions and avoid introducing temperature as an additional source of variation between the species. Females are primarily responsible for brood construction and provisioning, while male participation influences dung relocation efficiency and reproductive output. Accordingly, experimental treatments manipulated beetle density and their sex ratio to reflect biologically relevant breeding units.

### 2.1 Field Collection and Sample Storage

Two species of dung beetles, viz. *O. binodis* and *D. gazella*, were selected for the current study. They were chosen because both species are abundant in the study area. They were collected from the pastures of Farrer Memorial Agricultural High School (Latitude 31°08′22″S, Longitude 150°58′53″), Calala, Tamworth, New South Wales. The beetles were collected using fresh cattle dung‐baited traps (November 2022) and hand‐collected during each site visit. All collected beetles were taken to the insect ecology laboratory, cleaned, and identified to species and sex under a stereomicroscope (Leica M60) using species‐specific morphological characteristics. In *O. binodis* , males and females were distinguished based on pronotal morphology, with major males having a conspicuous pronotal horn and females having a smaller, rounded pronotal projection. Minor males have greatly reduced or rudimentary horns; therefore, only major males were used in the experiment. In *D. gazella*, males were distinguished by paired posterior head horns, which are absent in females.

Fresh dung was collected from the same pastureland. Dung was collected in the morning (~9 a.m.) to avoid fly infestation. The cattle had not been treated previously with insecticides or anthelmintics and were fed green grass while being housed on pastureland. Dung pats were collected from the field using a hand trowel, put in 15‐L plastic buckets, and brought to the laboratory. Approximately 60 kg of dung was collected per visit in the field. After returning from the field, all dung was thoroughly mixed and homogenised into a single batch to minimise variation in dung quality among experimental replicates and to provide standardised resource across treatments. The purpose of homogenisation was to control variation in dung quality because the experiment was designed to test the effects of beetle density and sex ratio, rather than differences among individual dung pats. The homogenised dung was then divided into 20 plastic bags (~3 L each) and stored at −20°C for mass rearing and subsequent experimental trials. Dung was frozen at −20°C for at least 30 days as a standardised pre‐treatment to minimise the risk of mite and parasite infestation and to facilitate standardised storage. Before use, frozen dung was thawed at room temperature and homogenised again to provide a uniform resource for all experimental containers. Although freezing may alter some dung characteristics (Shine et al. 2022), all experimental containers received the same frozen–thawed dung, thereby reducing potential variation associated with dung quality. Representative dung samples from each 3 L bag were pooled, and the pH was measured using a calibrated digital pH meter (SKU: EC‐PH6/02 K; EcoScan Portable pH Meter; Charleston, South Carolina, USA). Approximately 500 g of the pooled samples were dried in a forced‐air oven at 60°C until a constant weight was achieved, following the AOAC protocol (AOAC International 2012). The dried samples were ground using an electric cyclone rotor mill (Retsch‐Allee 1‐5, Haan, Germany) fitted with a 0.5 mm sieve. Processed samples were sent to the Feed Central Laboratory (Warrego Hwy, Charlton, QLD 4350, Australia) for wet‐chemistry and mineral analysis. The chemical and mineral composition of cattle dung fed to the beetles is shown in Table 1.

**TABLE 1:** The chemical and mineral composition (expressed as g/kg dry matter unless specified otherwise) of cattle dung provided to dung beetles under controlled laboratory conditions.

Item	Dung
Chemical composition	
Moisture (g/kg)	879
pH	7.21
Dry matter (g/kg)	121
Organic matter	850
Crude protein	166
Neutral detergent fibre	500
Acid detergent fibre	324
Hemicellulose	176
Ash	150
Total nitrogen (N)	26.6
Total carbon (C)	425
C: N	16.0
Mineral composition	
Potassium	5.50
Calcium	34.6
Magnesium	11.3
Phosphorus	6.70
Sodium	1.30
Copper (ppm)	36.0
Iron (ppm)	854
Manganese (ppm)	179
Molybdenum (ppm)	1.20
Zinc (ppm)	112

### 2.2 Mass Rearing of Wild Beetles

To ensure that the beetles used in experimental observations were of a similar age and reproductive status, we used F1 beetles. Field‐collected beetles were reared in 9 L containers containing a mixture of sand and vermiculite (1:2). The substrate moisture content was standardised at approximately 10% at the beginning of the experiment using an ICT International Moisture probe (MPM160‐B, Toowoomba, Australia) under controlled laboratory conditions. This moisture level was selected to provide suitable conditions for beetle burrowing and brood construction while maintaining the structural integrity of the substrate. Fresh dung (~ 350 g) was placed on top of the mixture in each container, and 10 beetle pairs were introduced to each container for brood production. Beetles were fed twice a week, with unused dung removed from each container at each feeding. Two weeks later, broods were collected from the containers, and 30 broods were placed in a 5 L (32 cm length × 22 cm width × 10 cm height) container with moistened vermiculite. The surviving beetles were kept in a clean container following the above process. The brood containers were watered occasionally using a spray bottle to moisten the vermiculite, and a tiny empty container was placed at the level of vermiculite. After 3 to 4 weeks, brood containers were checked daily to collect the F1 beetles that had emerged from the broods and had fallen into the tiny container as they traversed the brood container. The collected F1 beetles were transferred promptly into another 3 L container with a sand and vermiculite (1:2) mixture and cattle dung and were reared for 15 days to ensure reproductive maturity. Randomly selected F1 females (~ 15 days old) from both species were dissected and examined microscopically for ovarian and oocyte development as a gold standard to measure reproductive maturity across coleopteran systems (Figure 1). Mass rearing was performed for both species in separate containers, maintaining the same procedure. Finally, the experiments were started with the sexually mature F1 beetles (average body weight of 111 ± 29.8 mg and 80 ± 20.1 mg (mean ± SD) for *O. binodis* and *D. gazella*, respectively). Because *O. binodis* is naturally larger than *D. gazella*, body weight could not be completely standardised between species. To minimise within‐species variation associated with body size (Cook, 1993, Chen et al. 2024), only major males of *O. binodis* and relatively large‐sized beetles of both species were used in this experiment. Enlarged pronotal horns on major males of *O. binodis* are employed in competition with other individuals to gain entry to female breeding chambers, which aid in developing broods (Simmons and Kotiaho 2007). Competition for dung is significantly influenced by the size or weight of the beetles; large‐sized tunnellers remove the dung quickly from the pat (Doube 1990).

**FIGURE 1:**
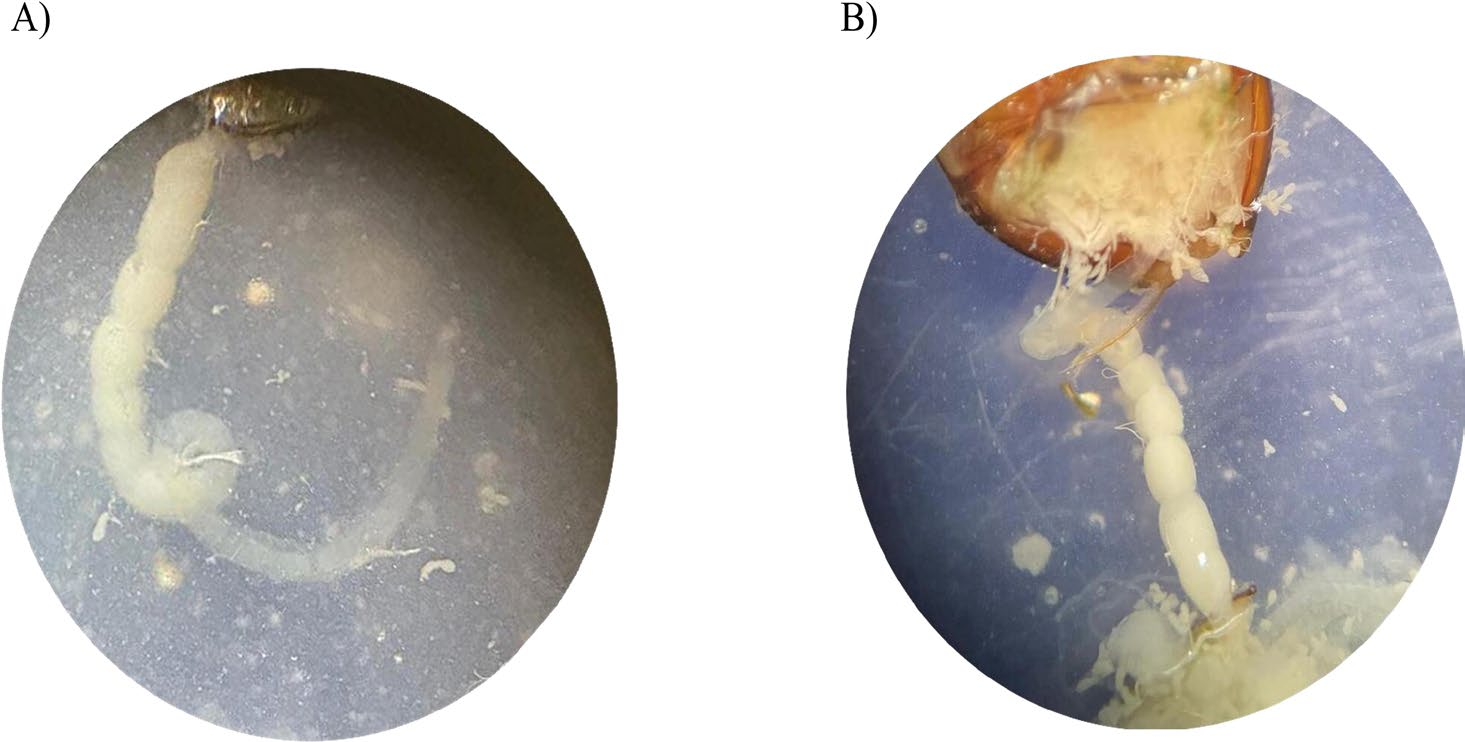
Dissections of sexually mature F1 female dung beetles: (A) *Onthophagus binodis* and (B) *Digitonthophagus gazella*, with exposed eggs observed under a light microscope. The images demonstrate reproductive maturity, revealing developed eggs within the ovaries of each species.

### 2.3 Experimental Design

The experiment ran from early January to June 2023 to determine the combined breeding density and sex ratio effect on intraspecific competition of dung beetles, including dung burial, brood production, brood size and brood dry weight (Figure 2). Experiments for *O. binodis* and *D. gazella* were conducted concurrently using F1 beetles and maintained under identical laboratory conditions. For each species, 210 sexually mature F1 beetles (120 females and 90 males) were randomly allocated among 3 L plastic containers according to the treatment combinations described below. Each container was filled with sand and vermiculite (1:2) to approximately 3 cm from each container's top. The moisture content of the nesting materials was maintained at approximately 10% across treatments. The beetles were reared for 14 days and fed 100 g of cattle dung twice weekly, allowing feeding and brood production. The same quantity of dung was provided to each experimental unit, thereby standardising total resource availability across treatments while reducing per‐capita resource availability as beetle density increased. (Gotcha et al. 2022). The unused dung in each experimental container was collected while feeding, carefully inspected for the attachment of any live beetles, weighed separately, and kept in the laboratory for later analysis. To avoid dung source interference, we collected cattle dung from a single pastureland (see field collection and sample storage section) and used it as feed for both species throughout the experiment.

**FIGURE 2:**
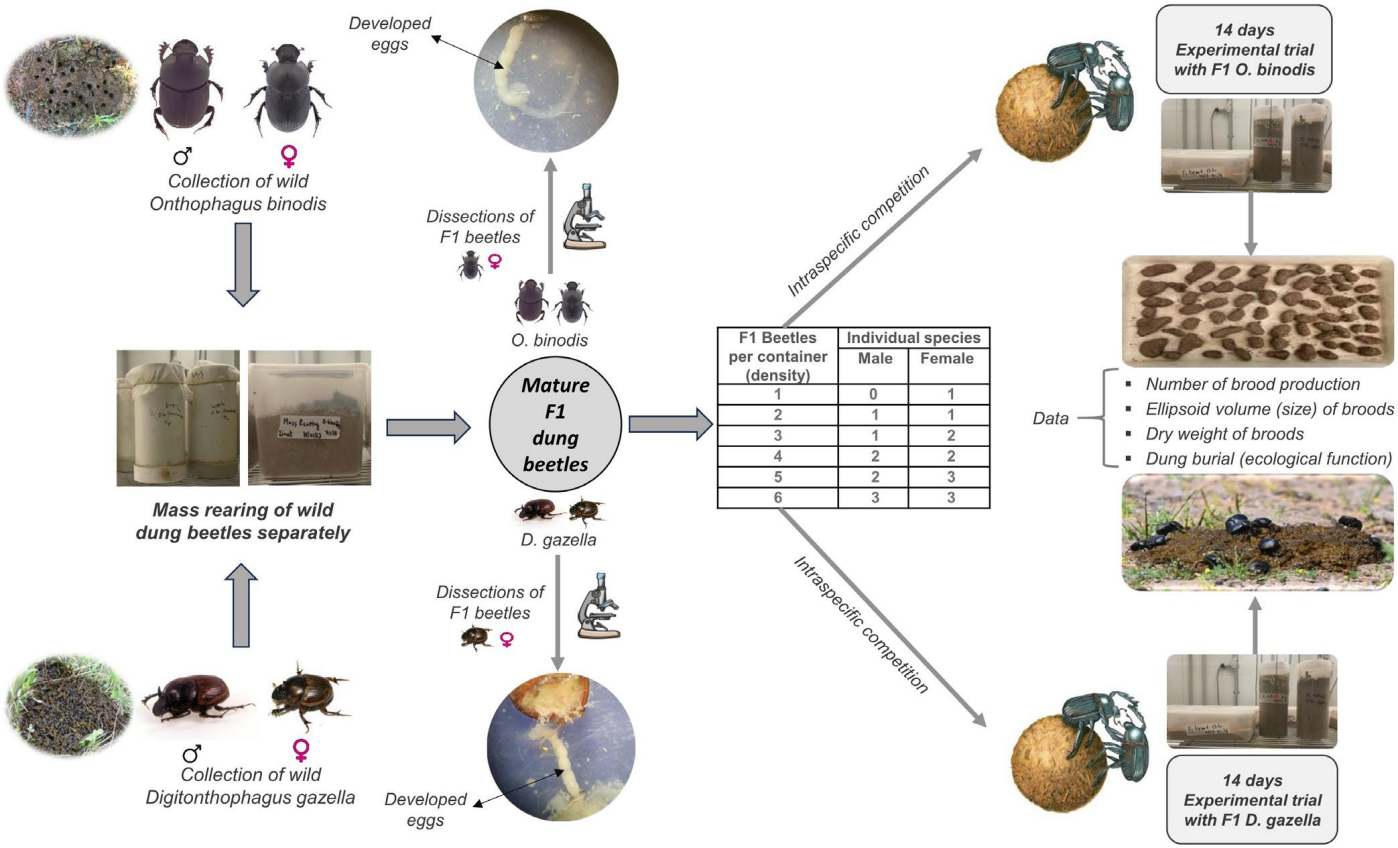
Schematic representation of the experimental design used to examine density‐ and sex ratio‐dependent reproductive performance and dung burial by *Onthophagus binodis* and *Digitonthophagus gazella*. Wild‐collected beetles were mass‐reared to obtain F1 adults, which were assigned to a gradient of density treatments. Reproductive output (brood production, brood size, brood dry weight) and dung burial were quantified after a 14‐day experimental period.

### 2.4 Treatments

To examine the effects of intraspecific density and sex ratio on dung burial, brood number, brood size and brood weight, the experiment was conducted separately for *O. binodis* and *D. gazella* using reproductively mature F1 beetles maintained under identical laboratory conditions. For each species, six treatment combinations were maintained following established approaches using low‐density and pair‐based designs in dung beetle experiments (Bornemissza 1970; Gotcha et al. 2022; Mamantov and Sheldon 2020; Zamprogna et al. 2025). The treatments comprised 1 female, 1 pair, 1 pair + 1 female, 2 pairs, 2 pairs + 1 female or 3 pairs, where each pair consisted of 1 male and 1 female (Table 2). Thus, the treatments represented increasing numbers of beetles and a different sex ratio within the same species.

**TABLE 2:** Experimental setup of male–female density combinations for both *O. binodis* and *D. gazella*.

Number of individuals per container	Treatments (density and sex ratio)	Number of male beetles	Number of female beetles
1	1 female	0	1
2	1 pair	1	1
3	1 pair + 1 female	1	2
4	2 pairs	2	2
5	2 pairs + 1 female	2	3
6	3 pairs	3	3

*Note:* Each pair = 1 male + 1 female.

For each species, the experiment was conducted using a randomised block design with 10 blocks. Each block consisted of one complete set of all six treatments, which were established simultaneously under identical laboratory conditions and therefore represented an independent experimental replicate. Treatments were randomly assigned within each block, resulting in 10 independent replicates per treatment.

A solitary female was selected as a control to provide a baseline measure of reproductive output and dung burial in the absence of intraspecific interactions, rather than as a direct comparison with male‐inclusive treatments (Zamprogna et al. 2025). All experimental females were obtained from the same cohort of 15‐day‐old F1 beetles that had been reared with males before treatment allocation. Female reproductive maturity was confirmed by ovarian dissection, which revealed the presence of mature eggs (Figure 1). Although insemination was not confirmed for individual females, all females were reared under similar pre‐experimental conditions and had equivalent opportunities to mate before being randomly assigned to treatments; however, the willingness to mate could vary among solitary females. Comparable beetle densities and sex ratio within blocks were used for both species in this experiment.

### 2.5 Dung Burial, Brood Production, Ellipsoid Volume and Brood Dry Weight

Approximately 150 g of fresh dung was subsampled during the feeding time. The collected unused dung and subsampled fresh dung were then dried in a forced‐air oven at 65°C until a constant weight was achieved, following AOAC guidelines (AOAC International 2012). The dried weight of the dung was recorded and pooled weekly. Dung burial was calculated by determining the organic matter (OM) content of pooled unused and fresh dung using the loss‐on‐ignition method (Bensharada et al. 2022; Heiri et al. 2001). In total, 120 unused dung samples (6 treatments × 2 week feeding period × 10 blocks/replicates) and 40 subsamples of fresh dung (2 subsamples × 2 week feeding period × 10 blocks/replicates) were analysed to determine their OM content using the following formula:

$$\mathrm{OM}\;\left(g\;\mathrm{per}\;\mathrm{kg},\mathrm{dry}\;\text{matter}\right)=1000-\left(\frac{\mathrm{ash}\;\text{weight}}{\mathrm{dry}\;\text{sample weight}}\times 1000\right)$$

Ash weight was determined by ignition of dried dung in a muffle furnace at 650°C for 8 h, following AOAC guidelines (AOAC International 2012). Dung burial was calculated based on the OM content of unused and fresh dung on a dry matter basis using the following formula:

$$\text{Dung buried}\;\left(g/\mathrm{kg}\;\mathrm{dry}\;\text{matter}\right)=\text{Fresh dung}\;\mathrm{OM}-\text{Unused dung}\;\mathrm{OM}$$

Reproductive investment was assessed by measuring brood number, brood dry weight and brood size, allowing us to evaluate how increasing density and sex ratio influenced allocation to reproduction under experimental conditions. After completing a 14‐day feeding trial, the total number of broods produced in each treatment container was counted. Each brood produced within an experimental container was collected separately, oven‐dried at 65°C for 7 days to achieve a constant weight. The dry weight of the brood was subsequently measured using an electronic balance. Afterwards, the ellipsoid volume (brood size) of each brood produced in the treatment container was calculated by measuring the equatorial and 2 polar diameters using digital vernier callipers (Kincrome 0–150 mm, Australia; precision ±0.01 mm) as described by Heddle et al. (2024). Each measured diameter was halved to obtain the corresponding radius. The ellipsoid volume was then determined using the following formula:

$$\text{Ellipsoid volume}\;\left(mm^{3}\right)=\frac{4}{3}\text{πabc}$$

where *a* is the equatorial radius, *b* is the first polar radius and *c* is the second polar radius.

### 2.6 Statistical Analyses

All statistical analyses were conducted using R‐Studio 4.4.3 (R Core Team 2022). Generalised linear mixed‐effect models (GLMMs) were fitted with the *‘glmmTMB’* package (Magnusson et al. 2017). Brood volume and brood dry weight were analysed using *Gamma* error distributions with a log link because these variables were continuous, strictly positive and right‐skewed. Residual diagnostics, including assessments for overdispersion and underdispersion, were performed using the simulateResiduals and testResiduals functions within the *‘DHARMa’* package (Hartig and Hartig 2017), confirming appropriate model fit with no evidence of non‐uniformity, dispersion, zero inflation, or outliers. To satisfy assumptions of residual uniformity, dispersion and outlier diagnostics, brood production was analysed using *Gaussian* GLMMs. Type III chi‐square tests were applied to the analysis of deviance table, and all pairwise comparisons were adjusted using the Holm‐Bonferroni method via the emmeans function from the *‘emmeans’* package (Lenth et al. 2019). Observed response distributions were visualised using boxplots generated with the *‘ggplot2’* package, while estimated marginal means from the fitted models were used for post hoc comparisons. Significant differences (*p* < 0.05) were denoted by distinct letters within the plots.

The parametric data, such as dung burial, were analysed using the following mixed model fitted with the lmer function from the *‘lme4’* package to evaluate the combined effect of beetle density and sex ratio for both feeding weeks.

$$Y_{\text{ijkl}}=\mu +D_{i}+W_{j}+\left(D_{i}\times W_{j}\right)+b_{k}+\epsilon_{\mathrm{ijk}}$$

where $Y_{\mathrm{ijk}}$ represents the response variable; μ is the overall mean; $D_{i}$ is the density effect of each species; $W_{j}$ represents the effect of the feeding period (weeks 1 and/or 2); $D_{i}\times W_{j}$ is the density‐by‐feeding period interaction; $b_{k}$ represents the effect of block, respectively; and $\epsilon_{\mathrm{ijk}}$ is the residual error term. The treatment density and sex ratio, feeding periods and their interactions were considered as fixed effects in the fitted model, while block was included as a random effect. Model assumptions of normality and homoscedasticity were assessed using residual and Q–Q plots and were found to be satisfactorily met; therefore, no transformation was applied.

For comparisons between *O. binodis* and *D. gazella*, block was treated as a random effect, with species and treatment included as fixed effects. The relationship between brood ellipsoid volume (response variable) and brood dry weight or brood number (predictor variables) was analysed using linear mixed‐effects regression models fitted with the lmer function in the *‘lme4’* package, incorporating block as a random effect to account for potential variability among experimental units. Our objective was to quantify the overall relationship between brood size and brood investment; therefore, no interaction terms were included. Fitted regression models yielded regression equations, marginal *R* ^2^, representing the variance explained by the fixed effects, and *p*‐values to assess the strength and significance of these relationships. All statistical models, including R code, model summary outputs, *DHARMa* residual diagnostics and diagnostic plots, are available at the figshare repository (Chowdhury et al. 2026; https://doi.org/10.6084/m9.figshare.31989726.v3).

## 3 Results

### 3.1 Brood Production

A total of 804 broods were produced across the treatments with *O. binodis*. We found that the combined effects of beetle density and sex ratio significantly influenced brood production (*χ* ^2^ = 22.9, *p* < 0.001) in *O. binodis* (Figure 3A). Brood production was highest when the density and sex ratio were 1 pair + 1 female or 2 pairs with 1 female, and lowest in the solitary female treatment of *O. binodis* . Other treatments showed intermediate levels of brood production.

**FIGURE 3:**
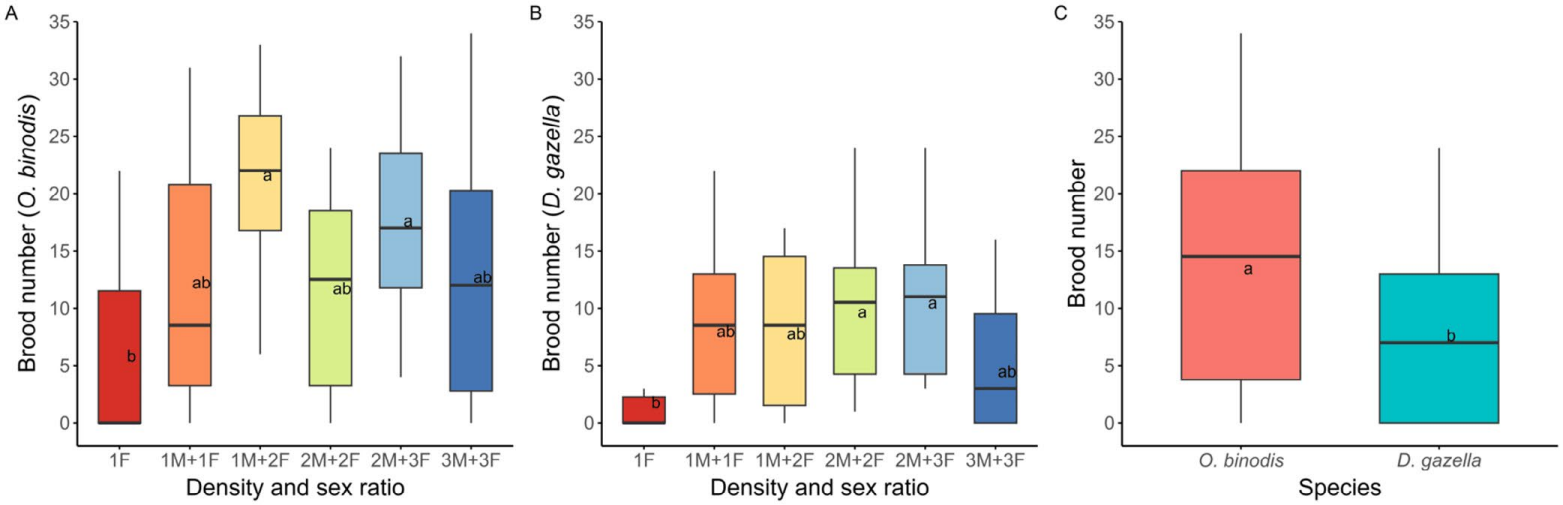
Combined effects of dung beetle density and sex ratio on brood production. (A) Brood number of *Onthophagus binodis* under different density and sex ratio treatments: Single female (1F), one male with one female (1 M + 1F), one male with two females (1 M + 2F), two males with two females (2 M + 2F), two males with three females (2 M + 3F) and three males with three females (3 M + 3F). (B) Brood number of *Digitonthophagus gazella* under the same density and sex ratio treatments. (C) Comparison of brood number between *O. binodis* and *D. gazella*, averaged across all treatments. Different letters within each boxplot indicate statistically significant differences (*p* < 0.05). Each treatment consisted of *n* = 10 independent experimental units.

On the other hand, 453 broods were produced across all treatments with *D. gazella*. It was found that brood production was significantly influenced by the combined effects of density and sex ratio (*χ* ^2^ = 19.6, *p* = 0.001) (Figure 3B). The maximum number of broods produced by *D. gazella* occurred when the density and sex ratio were maintained at 2 pairs or 2 pairs with 1 female. The lowest number of broods was produced in the case where there was 1 female. In addition, we observed that species identity strongly influenced brood production. *O. binodis* produced a higher number of broods (*p* < 0.001) than *D. gazella* (Figure 3C).

### 3.2 Ellipsoid Volume of Broods

We measured the ellipsoid volume of 804 *O. binodis* and 453 *D. gazella* broods, respectively. The ellipsoid volume was affected by the treatment (for *O. binodis* , *χ* ^2^ = 84.2, *p* < 0.001; for *D. gazella*, *χ* ^2^ = 28.3, *p* < 0.001). For instance, *O. binodis* produced larger broods (*p* < 0.001) under the following beetle densities and sex ratios: 1 female, 1 pair, 1 pair +1 female or 2 pairs, compared with 2 pairs with 1 female or 3 pairs (Figure 4A). On the other hand, *D. gazella* produced larger (*p* < 0.001) broods when the density and sex ratio was 1 pair or 1 pair with 1 female compared with 2 or 3 pairs (Figure 4B). In both species, smaller broods were formed in the highest beetle density with 3 pairs. However, we found that species greatly influence brood size. The bigger broods were formed by *D. gazella* compared to *O. binodis* (*p* < 0.001; Figure 4C).

**FIGURE 4:**
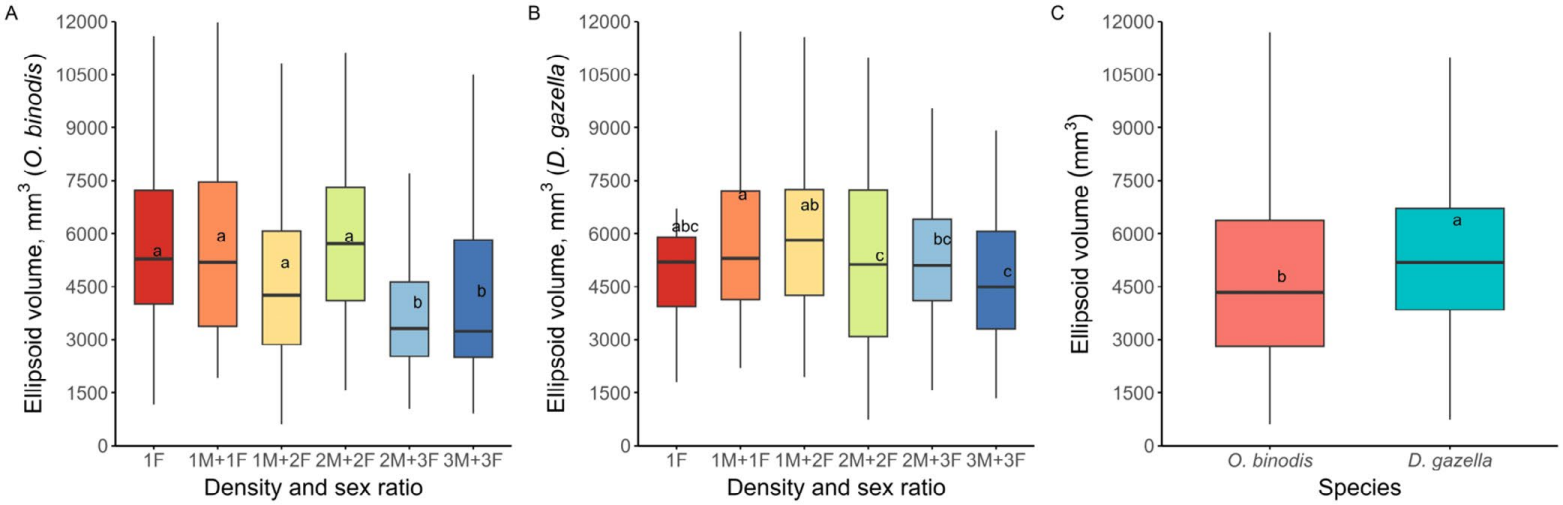
Combined effects of dung beetle density and sex ratio on brood ellipsoid volume. (A) Brood ellipsoid volume of *Onthophagus binodis* under different density and sex ratio treatments: Single female (1F), one male with one female (1 M + 1F), one male with two females (1 M + 2F), two males with two females (2 M + 2F), two males with three females (2 M + 3F) and three males with three females (3 M + 3F). (B) Brood ellipsoid volume of *Digitonthophagus gazella* under the same density and sex ratio treatments. (C) Comparison of brood ellipsoid volume between *O. binodis* and *D. gazella*, averaged across all treatments. Different letters within each boxplot indicate statistically significant differences (*p* < 0.05). Each treatment consisted of *n* = 10 independent experimental units.

### 3.3 Dry Weight of Broods

According to our findings, the combined effects of density and sex ratio significantly influenced the dry weight of broods of both species (*χ* ^2^ = 75.2, *p* < 0.001 for *O. binodis* ; *χ* ^2^ = 24.9, *p* < 0.001 for *D. gazella*). In the case of *O. binodis* , the brood's dry weight was highest (*p* < 0.001) in 1 or 2 pairs, followed by 1 female or 1 pair with 1 female, compared with 2 pairs with 1 female, which was lowest in weight (*p* < 0.001, Figure 5A). On the other hand, the brood dry weight of *D. gazella* was greater (*p* < 0.001) in 1 pair or 1 pair with 1 female contrasted with 2 or 3 pairs, whereas the remaining treatments had intermediate values (Figure 5B). The brood dry weight was species‐dependent, as *D. gazella* produced significantly heavier broods than *O. binodis* (*p* < 0.001; Figure 5C).

**FIGURE 5:**
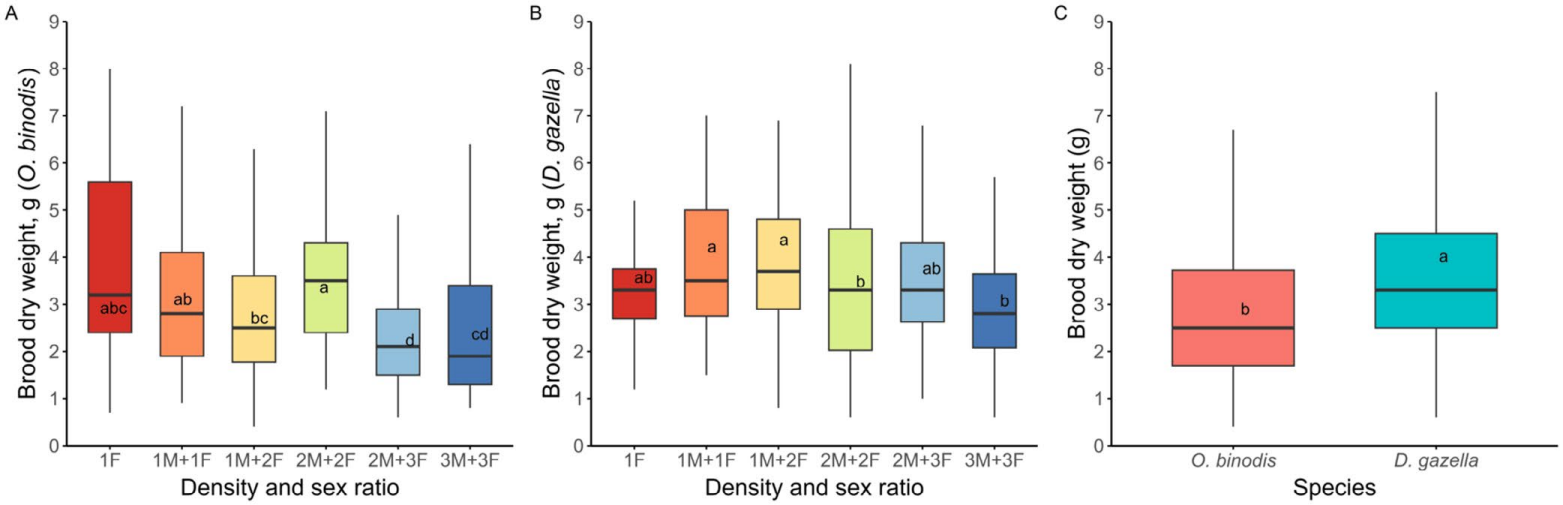
Combined effects of dung beetle density and sex ratio on brood dry weight. (A) Brood dry weight of *Onthophagus binodis* under different density and sex ratio treatments: Single female (1F), one male with one female (1 M + 1F), one male with two females (1 M + 2F), two males with two females (2 M + 2F), two males with three females (2 M + 3F) and three males with three females (3 M + 3F). (B) Brood dry weight of *Digitonthophagus gazella* under the same density and sex ratio treatments. (C) Comparison of brood dry weight between *O. binodis* and *D. gazella*, averaged across all treatments. Different letters within each boxplot indicate statistically significant differences (*p* < 0.05). Each treatment consisted of *n* = 10 independent experimental units.

### 3.4 Relationship Between Brood Size, Number and Dry Weight

Linear regression analyses revealed a strong positive correlation between the brood dry weight of both species and ellipsoid volume. In *O. binodis* , the relationship was highly significant (Ellipsoid volume = 395.84 + 1589.6 × Brood dry weight, *R* ^2^ = 0.766, *p* < 0.001), indicating that increases in brood dry weight were associated with larger ellipsoid volumes (Figure 6A). Similarly, a significant positive correlation (Ellipsoid volume = 227.04 + 1565.6 × Brood dry weight, *R* ^2^ = 0.792, *p* < 0.001) was observed in *D. gazella* (Figure 6B). We also observed a distinct relationship between brood number and ellipsoid volume in *O. binodis* . For *O. binodis* , a significant negative correlation was observed (Ellipsoid volume = −81.3 × Brood number + 6427.8, *R* ^2^ = 0.057, *p* < 0.001) between brood number and brood size; however, the low explanatory power indicates a weak relationship (Figure 7A). Conversely, for *D. gazella*, no significant correlation was detected (Ellipsoid volume = 36.6 × Brood number + 5737.5, *R* ^2^ = 0.005, *p* = 0.213), suggesting that ellipsoid volume remained consistent across brood numbers (Figure 7B).

**FIGURE 6:**
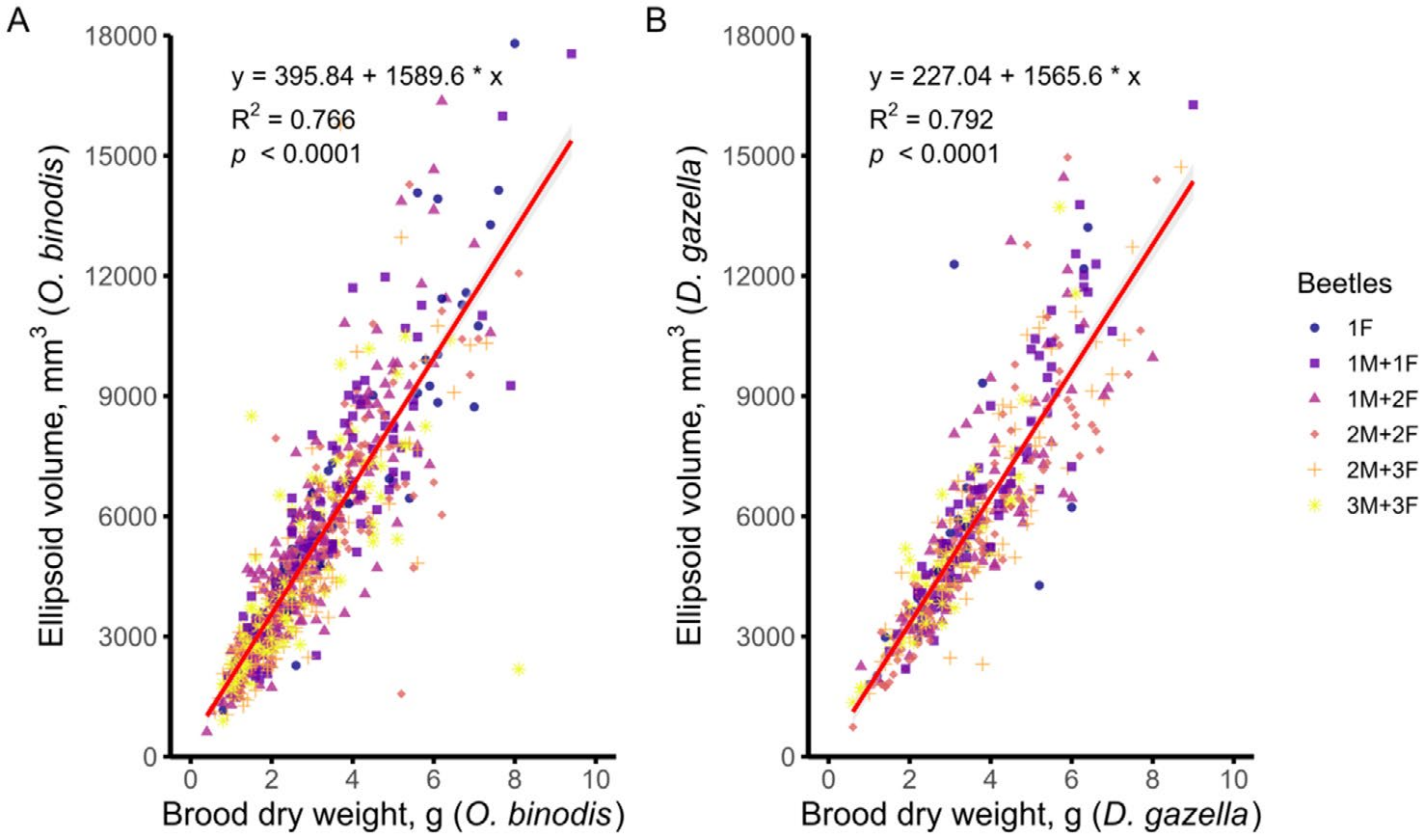
Relationship between brood dry weight and ellipsoid volume of dung beetle species *Onthophagus binodis* (A) and *Digitonthophagus gazella* (B) under varying densities and sex ratio. Each point represents an individual brood, colour‐coded by treatment densities and sex ratio: Single female (1F), one male with one female (1 M + 1F), one male with two females (1 M + 2F), two males with two females (2 M + 2F), two males with three females (2 M + 3F) and three males with three females (3 M + 3F). Each treatment consisted of *n* = 10 independent experimental units. A linear regression line was fitted with a regression equation, marginal *R* ^2^ and *p* values.

**FIGURE 7:**
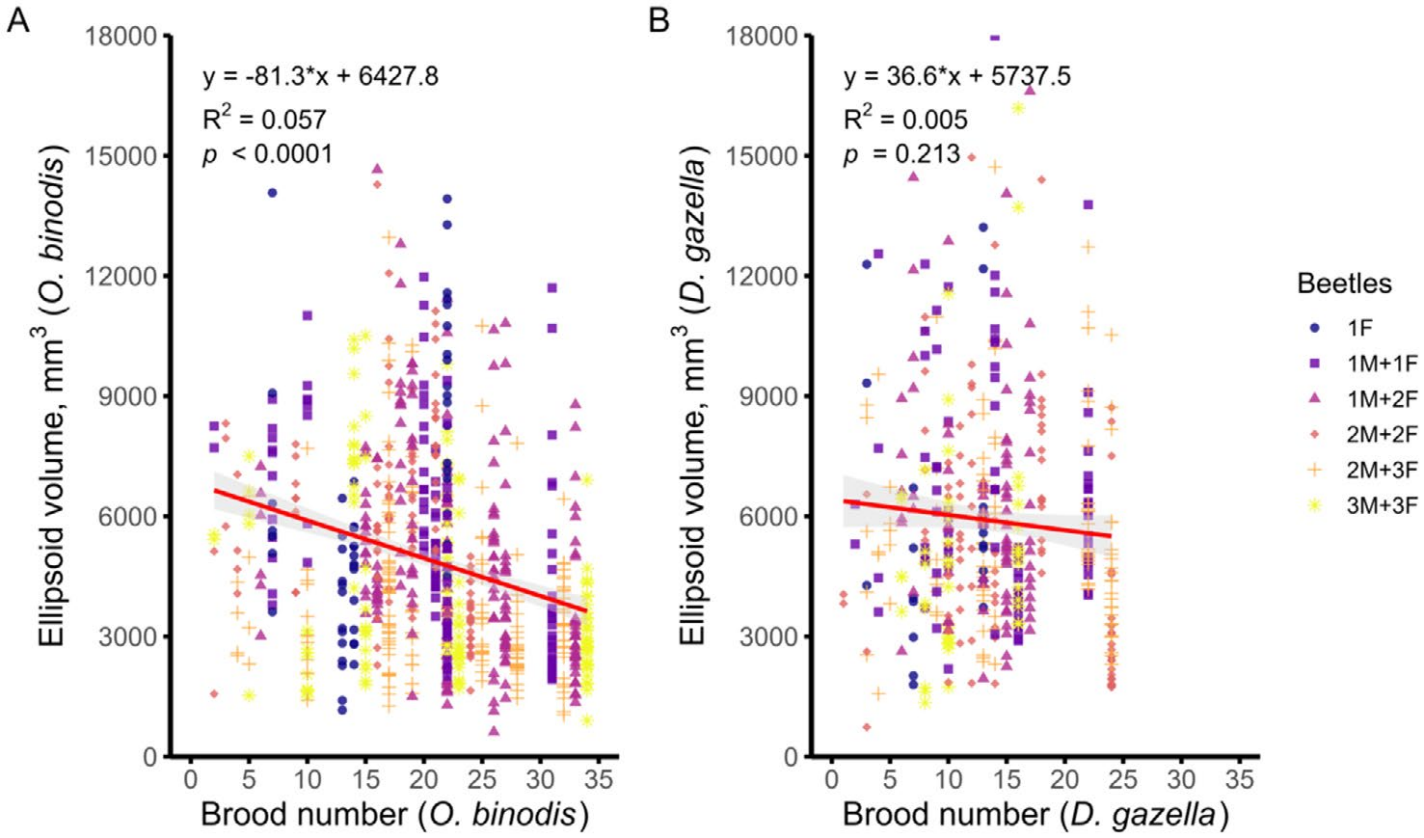
Relationship between brood number and ellipsoid volume of dung beetle species *Onthophagus binodis* (A) and *Digitonthophagus gazella* (B) under varying densities and sex ratio. Each point represents an individual brood, colour‐coded by treatment densities and sex ratio: Single female (1F), one male with one female (1 M + 1F), one male with two females (1 M + 2F), two males with two females (2 M + 2F), two males with three females (2 M + 3F) and three males with three females (3 M + 3F). Each treatment consisted of *n* = 10 independent experimental units. The solid line represents a fitted regression model, with the regression equation, marginal *R* ^2^ and *p* values.

### 3.5 Dung Burial

The combined effects of beetle density and sex ratio significantly (*p* < 0.05) influenced the dung burial in both species (Figure 8). However, the feeding period or the interaction between treatment and feeding period had no effect (*p* > 0.05) on dung burial. Dung burial was 91.5 g/kg dry matter higher (*p* = 0.003) in *O. binodis*, while the beetle density and sex ratio were maintained at 2 pairs with 1 female or 3 pairs compared to an individual female, with other treatments having intermediate values (Figure 8A). Similarly, dung burial was 124 g/kg dry matter higher (*p* < 0.001) in *D. gazella*, while the beetle density and sex ratio were 2 pairs or beyond that level compared to a solitary female, with other treatments having intermediate values (Figure 8B). Across all treatments, *D. gazella* buried significantly more dung than *O. binodis* (*p* < 0.001, Figure 8C).

**FIGURE 8:**
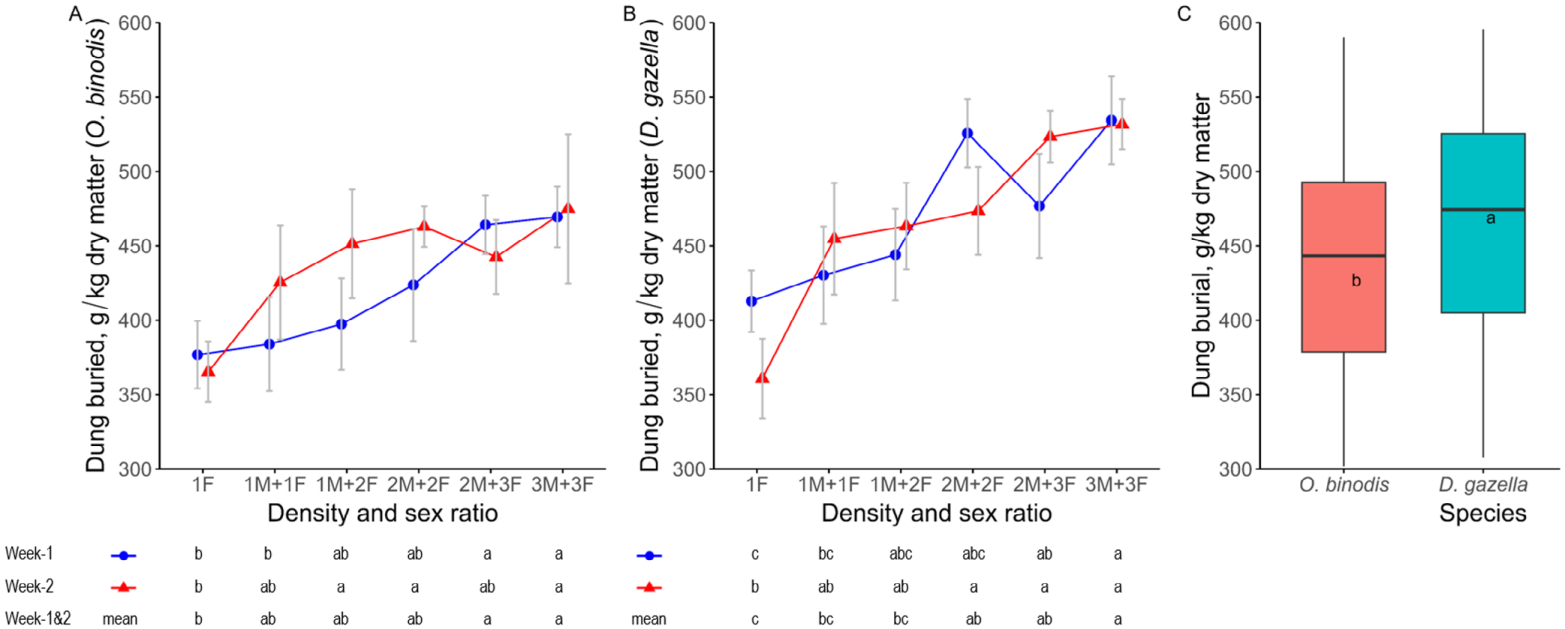
Combined effects of dung beetle density and sex ratio on dung burial. (A) Dung buried by *Onthophagus binodis* under different density and sex ratio treatments: Single female (1F), one male with one female (1 M + 1F), one male with two females (1 M + 2F), two males with two females (2 M + 2F), two males with three females (2 M + 3F) and three males with three females (3 M + 3F). Different letters below the x‐axis title indicate statistically significant differences (*p* < 0.05) for feeding period week 1, week 2 and both (week 1 and 2). (B) Dung was buried by *Digitonthophagus gazella* under the same density and sex ratio treatments. (C) Comparison of total dung buried between *O. binodis* and *D. gazella*, averaged across all treatments. Different letters within each boxplot indicate statistically significant differences (*p* < 0.05). Each treatment consisted of *n* = 10 independent experimental units.

## 4 Discussion

Organism density influences reproductive traits when individuals compete for ephemeral and spatially limited resources, such as mammalian dung (Ridsdill‐Smith et al. 1982; Giller and Doube 1989). Our results demonstrate that increasing beetle density combined with changes in sex ratio affected reproductive investment in both *O. binodis* and *D. gazella*, leading to greater dung burial that was accompanied by the production of smaller or lighter broods. However, the highest brood number was observed at intermediate treatment combinations (2 pairs with an additional female in both species, or 1 pair +1 female or 2 pairs in *O. binodis* and *D. gazella*, respectively). These findings suggest that the experimental treatments were associated with changes in resource allocation between brood number and per‐brood investment. Similar patterns have been observed under controlled laboratory conditions for *D. gazella* collected in Mexico, where this invasive species was shown to coexist with *Euoniticellus intermedius* through flexible reproductive strategies rather than complete competitive exclusion (Esquivel‐Román et al. 2023). Together, these findings suggest that reproductive decisions in tunnelling dung beetles were associated with the combined effects of density, sex ratio, and resource sharing under controlled conditions, while behavioural and reproductive plasticity may also facilitate coexistence even under increasing competitive pressure (Giller and Doube 1994), although behavioural mechanisms were not directly examined in the present study.

Our study showed that brood production was greatest in treatments containing 1 pair with 1 female or 2 pairs with 1 female for *O. binodis* and 2 pairs or 2 pairs with 1 female for *D. gazella*. These patterns suggest that reproductive output was influenced by the combined effects of beetle density and sex ratio in both species rather than density alone, as tested under current laboratory conditions. In addition, treatments containing more females (1 male vs. 2 females or 2 males vs. 3 females) generally produced more broods, indicating that female availability, together with the competitive environment, contributes to reproductive investment (Giller and Doube 1989; Gotcha et al. 2022; Mamantov and Sheldon 2023). Most of the published research related to competition in previous studies was conducted with wild beetles, and the age of the beetles was unknown when collected from the field. Giller and Doube (1989) found that brood production of the tunneller *Onitis alexis* (Klug) increased at lower density (8 pairs) and decreased with higher density (32 pairs). The difference in beetle density (8 pairs vs. 32 pairs) used by Giller and Doube (1989) was substantial; increased density led to intense competition between beetles for dung consumption, which increased the proportion of dung utilisation (70% to 80%) and contributed to significant differences in brood production, while our study used up to 3 pairs of beetles for both species. Additionally, their study used wild *O. alexis* beetles of unknown age, while ours controlled for age using laboratory‐reared F1 individuals.

*O. binodis* and *D. gazella*, across treatments, consumed only 50% to 55% of the dung OM, suggesting that dung availability was not fully exhausted during the experimental period under standardised laboratory conditions. Although a substantial proportion of dung remained unburied, competitive interactions between beetles may nevertheless have occurred through competition for access to dung resources, tunnelling and brood construction, rather than through complete source depletion alone. However, the observed proportion of dung consumption reflects outcomes under standardised laboratory provisioning and the relatively conservative density range examined and therefore should not be directly extrapolated to competitive dynamics under natural field conditions. In addition, beetles at higher densities of both species consumed more dung than in the control, which could lead to differences in brood construction. Our results are also aligned with the findings of Giller and Doube (1989), who reported no strong intraspecific competition in *Onitis* mixed species when the beetle density was lower than 4 pairs per dung pat. Although no mixed‐species treatments were included in our study, the observed density‐dependent responses in both species are comparable to laboratory studies demonstrating coexistence mechanisms under controlled competitive conditions (Esquivel‐Román et al. 2023). However, the density range tested in the current experiment was conservative relative to natural dung beetle populations. Further intra‐ and interspecific studies are needed to examine the effect of higher densities and sex ratio on dung burial and reproductive trade‐offs in both laboratory and field conditions.

When beetle densities of *O. binodis* were lower, brood production remained relatively constant over time, and the rate decreased during higher initial beetle densities (Ridsdill‐Smith et al. 1982). Ridsdill‐Smith et al. (1982) collected broods weekly, and densities were varied from 2 to 100 beetles. In addition, Ridsdill‐Smith et al. (1982) added some beetles at a higher density and found that brood production increased in subsequent weeks. The variation in brood production could be due to the mixing of wild and laboratory‐reared beetles and increasing the beetle population at higher density, which may disrupt the results of brood production. However, our study was not designed to mix wild beetles with F1 individuals. Hirschberger (1998) showed that *Aphodius ater* females laid a small number of eggs with increasing density. Also, different dung types may affect the brood production of dung beetles (Arellano et al. 2015). Hunt and Simmons (2004) found that female *Onthophagus taurus* produce more broods in horse dung compared with cattle dung. Our objective was not to investigate dung type, but rather to consistently use cattle dung throughout the trial from the same pastureland, and the dung was homogenised before use. We note that dung quality (moisture, protein and macro elements present in dung) and seasonal variation (Kaur et al. 2021) may influence brood production in dung beetles and warrant further investigation.

We observed that *O. binodis* produced more broods compared to *D. gazella*. Both species are tunnellers, but the size or body weight of the beetles used in this study was not similar. Therefore, the observed differences in brood production may partly be attributed to variation in the size of the beetles as well as species‐specific reproductive characteristics. Similarly, Arellano et al. (2017) reported that both sexes of *Onthophagus lecontei* contributed to brood construction; however, the overall reproductive output was influenced by the size and body condition of the parental beetles. The size or body weight of our F1 *O. binodis* was comparatively higher (111 ± 29.8 mg vs. 80 ± 20.1 mg) than that of *D. gazella*; it is reasonable to assume that *O. binodis* buried a greater mass of dung than *D. gazella*, which contributes to producing a higher number of broods. Nervo et al. (2014) reported larger beetles were more efficient in dung utilisation than small beetles. For instance, Carvalho et al. (2018) observed that *Canthon smaragdulus* buried more dung than *Canthon sulcatus*, as *C. smaragdulus* is larger than *C. sulcatus*. However, under the present experimental conditions, *O. binodis* buried less dung while producing a greater number of smaller‐sized (i.e., ellipsoid volume) broods than *D. gazella*, indicating differences in brood allocation that cannot be explained by body size alone. We also observed a significant but weak negative relationship between brood ellipsoid volume and brood number in *O. binodis* (*R* ^2^ = 0.057), indicating that brood size is likely influenced by multiple interacting factors rather than brood number alone. There are many factors related to the brood size of dung beetles, such as dung beetle species, resource types, dung quality, seasonal variation and the cattle's pasture diet (Arellano et al. 2015; Heddle et al. 2024; Kaur et al. 2021; Ridsdill‐Smith et al. 1982). Our study indicated that smaller broods were produced in both species when the density and sex ratio were maximum (3 pairs), and conversely, the brood size increased when the density and sex ratio declined, which was anticipated. Under low density, female beetles may collect more dung for provisioning broods compared to higher densities (Hunt and Simmons 2000). On the other hand, under higher‐density treatments, beetles may have experienced greater resource limitation due to increased density together with reduced per‐capita resource availability, which may have contributed to the changes in brood number or brood size in both species. Kishi (2014) found that the brood size of the *Onthophagus atripennis* was positively correlated with maternal size; however, female body size also significantly affects survival and fertility, as larger females have greater potential fitness for reproduction (Hunt and Simmons 2002).

We found a positive correlation between ellipsoid volume and the dry weight of broods (Figure 6). Brood dry weight varied across treatments, with heavier broods generally produced under lower beetle densities and lighter broods at higher densities. This pattern is consistent with reduced per‐offspring investment under increasing density and altered sex ratio, although the underlying behavioural mechanisms were not measured directly. Our study found that an even density level (except for 3 pairs) produced the highest dry weight of broods in *O. binodis* . When a male–female pair occupies a nest, dung allocation could be increased with availability, which may have facilitated greater investment in individual broods, whereas treatments containing more and unbalanced beetles may have reduced resource allocation per individual (Doube and Marshall 2014; Finn and Gittings 2003). Despite a strong positive relationship between brood volume and brood dry weight in both species, *O. binodis* produced a greater number of smaller and lighter broods than those of *D. gazella*. This pattern is consistent with a reproductive trade‐off in which greater brood production is associated with reduced investment per offspring under increasing density and altered sex ratio (Simmons and Ridsdill‐Smith 2011). In dung beetles, similar density and resource‐mediated shifts in brood mass have been linked to constrained dung availability per reproductive event and differences in allocation strategies among species (Mamantov and Sheldon 2020). Together, these results indicate that the two species differed in how reproductive investment was allocated across the experimental treatments, with *O. binodis* tending to produce more but smaller broods than *D. gazella*.

A key ecological function of dung beetles is the burial or utilisation of dung, which occurs by creating tunnels. Across the experimental treatments, greater beetle abundance was generally associated with increased dung burial, which may reflect the contribution of more individuals to dung excavation and brood construction (Giller and Doube 1989). These findings suggest that there may be a relationship between the amount of dung burial and the number of broods produced. However, the relationship between dung burial and brood production was not strictly proportional, indicating that resource allocation varied among treatments. For instance, females in the control group consumed more OM and produced fewer broods (brood number/OM consumed = 0.010, data not shown) as they were solitary in their habitat. In addition, the contrasting responses of *O. binodis* and *D. gazella* further suggest that species‐specific behavioural traits or differences in reproductive allocation, rather than beetle abundance alone, may have contributed to dung burial under the standardised laboratory conditions. Therefore, the rate of dung burial can vary depending on the species, beetles' competitive scrambling and shared excavation of dung resources, laboratory conditions, beetles' physiological plasticity, or behavioural differences associated with major males.

Our findings should be interpreted within the context of several experimental limitations. The experiment was restricted by the combined manipulation of density and sex composition, equal dung provision across treatments (resulting in lower per‐capita food availability at higher densities), the use of two species that differed in body size, a solitary female as the control, selection of only major males, the conservative density range (maximum three breeding pairs per container), and controlled laboratory conditions. These factors should be considered when interpreting the findings and limit direct extrapolation to natural dung beetle populations. Therefore, future studies incorporating independent manipulation of density, sex ratio and resource availability across broader density ranges under laboratory and field conditions will help clarify the mechanisms underlying reproductive allocation and ecosystem functioning.

In summary, increasing density combined with sex ratio enhanced dung burial but reduced brood size and brood dry weight, indicating shifts in reproductive allocation under increasing intraspecific interactions. On the other hand, the inverse relationship between brood number and brood size is consistent with the expected trade‐off between brood quantity and per‐brood investment under experimental conditions. Compared with *D. gazella*, *O. binodis* produced more but smaller and lighter broods, suggesting differences in reproductive allocation between the two species rather than demonstrating differences in offspring quality or distinct life‐history strategies. These findings provide insights into reproductive allocation and dung burial under controlled laboratory conditions, but further field validation is needed to assess their relevance to natural ecosystems and pasture management.

## Author Contributions

**Zinat Jahan Chowdhury:** conceptualization (equal), data curation (equal), formal analysis (lead), investigation (lead), methodology (lead), project administration (equal), resources (equal), software (lead), visualization (lead), writing – original draft (lead). **Zac Hemmings:** conceptualization (equal), data curation (equal), formal analysis (equal), investigation (equal), project administration (equal), resources (equal), software (equal), supervision (equal), visualization (equal). **Alfonsina Arriaga‐Jimenez:** data curation (equal), investigation (equal), methodology (equal), project administration (equal), supervision (lead), validation (supporting), visualization (supporting), writing – review and editing (equal). **Karl Vernes:** funding acquisition (equal), project administration (equal), resources (equal), supervision (equal). **Timothy M. Schaerf:** data curation (lead), formal analysis (supporting), funding acquisition (supporting), project administration (equal), resources (equal), supervision (equal), writing – review and editing (lead). **Nigel R. Andrew:** conceptualization (lead), data curation (equal), funding acquisition (equal), investigation (equal), project administration (equal), resources (equal), supervision (lead), writing – review and editing (lead).

## Funding

The authors have nothing to report.

## Conflicts of Interest

The authors declare no conflicts of interest.

## Data Availability

Data used in this manuscript, including the R code for the statistical analyses, are available in the figshare digital repository https://doi.org/10.6084/m9.figshare.31989726.v3 (Chowdhury et al. 2026).
